# Synergistic approach of PCR-based fragment length analysis and amplicon deep sequencing reveals rich diversity of *S-*alleles in sweet cherries from the Caucasian region of origin

**DOI:** 10.3389/fpls.2024.1355977

**Published:** 2024-04-05

**Authors:** Susan Schröpfer, Mirko Schuster, José Quero-Garcia, Gregorio López-Ortega, Henryk Flachowsky

**Affiliations:** ^1^ Julius Kühn-Institut (JKI), Federal Research Centre for Cultivated Plants, Institute for Breeding Research on Fruit Crops, Dresden-Pillnitz, Germany; ^2^ UMR BFP, INRAE, University of Bordeaux, Villenaved’Ornon, France; ^3^ Atlantic Green S.L, Ctra. Almonte-El Rocío, Huelva, Spain

**Keywords:** *S*-alleles, sweet cherry, genotyping, diversity, amplicon-deep sequencing, fragment length analysis, Caucasus, *S*-allele reference collection

## Abstract

**Introduction:**

The self-incompatibility system in sweet cherry (*Prunus avium* L.) prevents fertilization with own or genetically related pollen, and is genetically determined by the multi-allelic *S*-locus. Therefore, determining *S*-alleles is crucial for plant breeding and fruit production, as it enables the selection of compatible combinations of *S*-genotypes for successful pollination.

**Methods:**

In this study, *S*-alleles were identified in a total of 260 genotypes from the Caucasian region, the species’ center of origin. *S*-allele genotyping was conducted using PCR fragment length analysis with the standard marker PaConsI-F/R2 and reference genotypes, complemented by sequence analysis through amplicon deep sequencing.

**Results and discussion:**

The genotypes collected from Azerbaijan and Turkey exhibit a high allelic richness at the *S*-locus, particularly compared to modern sweet cherry cultivars worldwide. Nine previously undescribed *S*-alleles were identified and designated as *S_45_*, *S_46_*, *S_47_*, *S_48_*, *S_49_*, *S_50_*, *S_51_*, *S_52_* and *S_53_*. Given the expected high diversity for other traits, this plant material represents a valuable resource for further breeding research and introgression of new traits in future breeding programs. Furthermore, our results underscore that fragment length alone may not be sufficient for unambiguous assignment of *S*-alleles due to minimal length differences between different alleles. To address this issue, an *S*-allele reference ladder was developed using the rich diversity for precise assignment of the *S*-alleles. This tool can be applied in future experiments as a robust and cost-effective method for accurate *S*-genotyping across different runs and laboratories. Additionally, several selected *S*-genotypes were planted in a trial field and will be maintained as an S-allele reference collection.

## 1 Introduction

Sweet cherry (*Prunus avium* L.) belongs to the most popular temperate fruit crops. Total global production of sweet cherry in 2018 was 2.6 million tons with a steady upward trend over the past 20 years (FAOSTAT; http://www.fao.org/faostat/en/#data/QC/visualize), highlighting its increasing agronomic and economic importance worldwide. In addition to its role as a cultivated fruit tree, wild *P. avium* trees coexist in the same geographic areas as an important component of natural forest ecosystems in Europe, Asia, and Africa (Pinosio et al., 2020).

The origin of modern sweet cherry cultivars is believed to be in countries south of the Caucasus around the Caspian and Black Seas. Birds probably drove the subsequent spread to Europe, which is considered the native territory of sweet cherry (Iezzoni et al., 2017; Dondini et al., 2018). It is known from archaeological findings that early inhabitants in Europe consumed *P. avium* as a wild fruit around 4,000 – 5,000 B.C. Domestication of sweet cherry probably occurred in the Danube Valley in the Neolithic period 4,000 years ago (Faust and Surányi, 1996). In the Roman period, sweet cherry cultivation was introduced to Europe, documented e.g. by Roman mosaics from the 3^rd^ century found in Germany (Faust and Surányi, 1996) and written references dating to 300 B.C (Brown et al., 1996). The importance of sweet cherry cultivation increased in the 16th century, with the most intensity in Central Europe (Watkins, 1976). At that time, various cultivars were already cultivated (Burger et al., 2011). In the 19^th^ century, early settlers from Europe introduced sweet cherries to the American continent (Brown et al., 1996; Faust and Surányi, 1996).

Little is known about the development of cherry cultivars in the Middle Ages. Cherries were likely cultivated in different regions of Europe, and thus a large number of landraces have been developed and adapted to their local conditions (Iezzoni et al., 1990; Hanke and Flachowsky, 2017). Many of these landraces were used in breeding programs and some are still used for local production. Targeted breeding of sweet cherry cultivars, nowadays practiced in many countries, is still a very young discipline and started at the beginning of the 19^th^ century (Iezzoni et al., 2017). From a genetic point of view, modern cultivars are only a few generations distant from their early ancestors (Iezzoni et al., 1990). Breeding of new sweet cherry cultivars with better properties is difficult because the genetic basis of this species is very limited (Hanke and Flachowsky, 2017; Iezzoni et al., 2017). Studies using single nucleotide polymorphism (SNP) markers to analyze the genetic diversity of wild accessions, landraces, and modern cultivars revealed a significant genome-wide loss of variation among the three groups (Pinosio et al., 2020). Two different, successive bottlenecks have led to less diversity. The first and largest decline in diversity occurred during domestication, and a later, minor bottleneck occurred because of breeding.

In order to expand the scope of breeding material in terms of diversity, collecting, characterizing, and evaluating genetic resources from areas expected to have high diversity is beneficial to achieve future breeding goals. In 1926, the Russian botanist Nikolai I. Vavilov described the Caucasus region as one of the centers of origin of cultivated plants (Vavilov, 1926). Since the diversity of the species of interest in such centers is very high, they are ideally suited as places for collecting trips to expand the breeding gene pool. In this work, seedlings of sweet cherry landraces and wild *P. avium* trees from two countries south of the Caucasus (Azerbaijan and Turkey) were genotyped. In Turkey, the collection area was located in the southern part of the Kaçkar Mountains. The vegetation there is restricted to the river and stream valleys, and vegetation-free mountain ridges form a natural barrier between different plant populations. In Azerbaijan, which is an important fruit-growing center in the Caucasus region with diverse agro-environmental conditions, the main fruit production areas are located in the Northwest part near Guba and Khachmaz. The natural distribution of sweet cherry in Azerbaijan was described along the tributaries of the Kur River in the center and in the Talish Mountains near Lankaran in the south (Grossgejm, 1952).

One important feature with implications for the cultivation and breeding of sweet cherry is self-incompatibility (SI), which prevents fertilization with own or genetically related pollen and ensures a constant genetic exchange within and between populations (De Nettancourt, 1984). In commercial sweet cherry production, a significant percentage of the cultivated area is required for genetically compatible pollinator genotypes (Eeraerts et al., 2019), and parameters such as e.g. flowering time, picking time, and distance to the main cultivar need to be considered for optimal orchard management. The mechanism of gametophytic self-incompatibility (GSI) in *Prunus* species is determined by a single highly polymorphic genetic locus, the so-called *S*-locus. This locus consists of two closely coupled *S*-genes responsible for self-recognition and self-rejection after pollination (Tao and Iezzoni, 2010). Genetic variants of the *S*-locus are referred to as ‘*S*-haplotypes’ or ‘*S*-alleles’ and typically, two different *S*-alleles are present in diploid sweet cherry genotypes. The pistil *S*-gene is specifically expressed in the diploid pistil tissue and encodes for an enzyme with ribonuclease activity, the S-RNase, which acts as cytotoxin in self-pollen tubes (Tao and Iezzoni, 2010). The pollen *S*-gene encodes for an S-haplotype-specific F-box protein (SFB) and is expressed in the haploid male gamete (Tao and Iezzoni, 2010). According to an actual model of *S-RNase*-based self-incompatibility, S-RNases are transmitted from the style to the cytoplasm of the growing pollen tube and there, a general undefined RNase inhibitor detoxifies them. It is assumed that SFB proteins from the same *S*-allele type protect S-RNases from degradation, resulting in pollen tube growth inhibition through RNA degradation by the S-RNase activity (Hua et al., 2008).

Cultivars with the same *S*-genotype are cross-incompatible and belong to the same incompatibility group (IG). PCR methods based on the detection of the length polymorphism of the *S-RNase* gene (Tao et al., 1999; Wiersma et al., 2001; Sonneveld et al., 2003, Sonneveld et al., 2006) and the *SFB* gene (Vaughan et al., 2008) have been developed for the determination of the different *S*-alleles. The improved consensus primer pair flanking the first intron of the *S*-*RNas*e gene, namely PaConsI-F/R2, was suggested for high throughput *S*-allele genotyping (Sonneveld et al., 2006). This system has become well-established and has been used extensively for *S*-allele genotyping of sweet cherry (Vaughan et al., 2008; Ganopoulos et al., 2012; Sharma et al., 2016; Cachi et al., 2017; Marchese et al., 2017; Azizi-Gannouni et al., 2018) and has also be transferred to other *Prunus* species (Halász et al., 2021a, Halász et al., 2021b; Nicolás-Almansa et al., 2023). It further served as a basis for the further development of the genotyping method, such as the one-tube reaction assay described by Cmejlova et al. (2023). Different *S-*alleles of sweet cherry were numbered consecutively and to date, 23 *S*-alleles were described in commercial sweet cherry cultivars (Schuster, 2020; Cmejlova et al., 2023). Further *S*-alleles have been identified in wild sweet cherries accessions (Cachi et al., 2017), which have not yet been found in cultivated cherries.

Although this system works well, it has two weaknesses. Firstly, the fragment lengths detected by different electrophoresis systems can vary slightly due to the differences in instrumentation and chemistry used. This makes it difficult to assign alleles accurately, especially if there are no standards with a known *S*-allele genotype for comparison. Secondly, since the assignment of *S*-alleles is based on the fragment length of amplicons, it is always uncertain whether two PCR fragments of the same length from two different genotypes also undoubtedly originate from the same *S*-allele.

In this study, seedlings of landraces and wild sweet cherry trees collected in two countries in the center of origin were investigated to (i) determine the diversity of *S*-alleles using a newly introduced *S*-allele reference ladder and amplicon deep sequencing and (ii) develop a reference set of genotypes representing the currently known *S*-alleles of sweet cherry.

## 2 Materials and methods

### 2.1 Plant material

Stones from local sweet cherry cultivars and wild sweet cherries were collected in 2008 in Turkey and 2014 in Azerbaijan, separated from the fruit flesh, air-dried and stored under dry conditions. The collection area in Turkey was located in the northeast at the southern part of the Kaçkar Mountains. In Azerbaijan, sampling was performed in the center of the country along the tributaries of the Kur river and in the Talish Mountains in the south. In both countries, stones from local sweet cherries and wild sweet cherries were collected. The stones were transferred to the Julius Kühn-Institute (JKI), Institute for Breeding Research on Fruit Crops (Dresden, Germany) and sown in soil in a greenhouse after four-month of stratification at 4°C in perlite. In the year following sowing, the trees obtained (one to ten seedlings per mother tree) were raised in a nursery and then planted on the experimental field in Dresden-Pillnitz (in 2010: collection of Turkish origin; in 2017 collection of Azerbaijan origin). The cultivars used as reference genotypes for *S*-allele genotyping (**Table 1**) were grown in the same experimental field except of ‘Talaguera Brilante’ and ‘Pico Colorado’. Leaf material of these cultivars was kindly provided by Ana Wünsch (Agrifood Research and Technology Centre of Aragón) for reference. Selected genotypes of reference set for *S-*allele genotyping (**Table 2**) were grafted on the root stock *Prunus avium* ‘Alkavo’ and planted in the experimental field in 2023.

**Table 1:** *S*-allele genotyping by fragment length analysis and amplicon deep sequencing.

*S*-allele	length (bp)				reference genotype^1^	sequence analysis			
	ABI	Beckman		Diff.		Seq-ID	best BLAST hit	ident. [%]	short description
***S*_31_**	203	208		5		OQ511575	JQ280529.1	100.0	*P. avium, S*-RNase-*S*_31_, partial cds
***S*_3_**	229	234		5	‘Nordwunder’ (*S*_3_ *S*_12_), ‘Fertard’ (*S*_3_ *S*_6_), ‘Alex’ (*S*_3_ *S*_3’_), ‘Sir Tom’ (*S*_3_ *S*_13_)	OQ511563	AJ635286	100.0	*P. avium*, *S*-RNase *S*_3_, exons 1-2
***S*_3’_**	229	234		5	‘Alex’ (*S*_3_ *S*_3’_)				
***S*_14_**	325	331		6	‘Ferminia’ (*S*_1_ *S*_14_), ‘Adriana’ (*S*_1_ *S*_14_)	OQ511568	DQ266445	100.0	*P. avium*, *S*-RNase *S*_14_, partial cds
***S*_45_**	334	340		6		OQ511581	KF975455	91.8	*P. armeniaca*, *S*-RNase *S*_53_, complete cds
***S*_29_**	335	340		5		OQ511573	DQ266441	99.7	*P. avium*, *S*-Rnase *S*_29_, partial cds
***S*_18_**	337	342		5		OQ511570	DQ983363 ^b^	97.6	*P. tenella*, *S*-RNase *S*_4_, partial cds
***S*_2_**	338	344		6	‘Feria’ (*S*_2_ *S*_4_), ‘Narana’ (*S*_2_ *S*_9_)	OQ511562	AJ635284	100.0	*P. avium*, *S*-RNase *S*_2_ precursor, exons 1-2
***S*_12_**	339	345	344^a^	6(5)	‘Nordwunder’ (*S*_3_ *S*_12_), ‘0900 Ziraat’ (*S*_3_ *S*_12_)				
***S*_7_**	341	n.d.			‘Benjaminler’ (*S*_1_ *S*_7_), ‘Pflugwirtle’ (*S*_7_ *S*_19_)				
***S*_46_**	344	350		6		OQ511582	GU968641	95.6	*P. dulcis*, *S*-RNase *S*_22_, partial cds
***S*_47_**	345	350		5		OQ511583	EU448294	95.9	*P. dulcis*, *S*-RNase *S*_31_, partial cds
***S*_9_**	351	357		6	‘Ferobi’ (*S*_6_ *S*_9_), ‘Grace Star’ (*S*_4’_ *S*_9_), ‘Narana’ (*S*_2_ *S*_9_)	OQ511567	AJ635271	100.0	*P. avium*, *S*-RNase *S*_9_, exons 1-2
***S*_48_**	360	365		5		OQ511584	MG712735	75.5	*P. armeniaca*, *S*-RNase *S*_52_, partial cds
***S*_36b3_**	363	368		5		OQ511579	EU042130	100.0	*P. cerasus*, *S*-RNase *S*_36b3_, complete cds
***S*_36b2_**	365	370	369^a^	5(4)		OQ511578	EU042129	100.0	*P. cerasus*, *S*-RNase *S*_36b2_ complete cds
***S*_21_**	369	n.d.			‘Talaguera Brilante’ (S_21_S_22_), ‘Maergelikirsche’ (S_5_S_21)_				
***S*_34_**	372	377	378^a^	5(6)		OQ511577	JQ280525	100.0	*P. avium, S*-RN*ase S*_34_, partial cds
***S*_1_**	374	380		6	‘Ferminia’ (*S*_1_ *S*_14_), ‘Adriana’ (*S*_1_ *S*_14_), ‘Basler Adlerkirsche’ (*S*_1_ *S*_5_), ‘Benjaminler’ (*S*_1_ *S*_7_), ‘Lapins’ (*S*_1_ *S*_4’_)	OQ511561	AJ635282	100.0	*P. avium*, *S*-RNase *S*_1_, exons 1-2
***S*_49_**	376	381	380^a^	5(4)		OQ511585	AJ635280	82.5	*P. avium*, *S*-RNase *S*_16_, exons 1-2
***S*_30_**	379	385		6		OQ511574	DQ266442	100.0	*P. avium*, *S*-RNase, *S*_30_ allele, partial cds
***S*_50_**	385	390		5		OQ511586	DQ058402	81.7	*P. dulcis*, *S*-RNase *S*_i_, partial cds
***S*_5’_**	386	392		6		OQ511565	EU077235	100.0	*P. avium*, *S*-RNase *S*_5_, cultivar ‘Kronio’, partial cds
***S*_5_**	387	393		6	‘Rita’ (*S*_5_ *S*_22_), ‘Basler Adlerkirsche’ (*S*_1_ *S*_5_), ‘Maergelikirsche’ (S_5_S_21)_				
***S*_51_**	388	394	393^a^	6(5)		OQ511587	AB364469	91.6	*P. mume*, *S*-RNase *S*_8_, partial cds
***S*_17_**	391	396		5		OQ511569	AM690354^b^	94.6	*P. webbii*, partial *S*-RNase *S*_n3_, exons 1-3
***S*_52_**	394	399		5		OQ511588	DQ790375	74.2	*P. salicina*, S-Rnase *S*_b_, partial cds
***S*_42_**	406	411		5		OQ511580	FN429354^b^	91.2	*P. dulcis*, S-Rnase *S*_23_, complete cds
***S*_32_**	410	416		6		OQ511576	EU516388^b^	100.0	*P. armeniaca*, *S*-RNase *S*_17_, partial cds
***S*_22_**	417	423	422^a^	6(5)	‘Talaguera Brilante’ (S_21_S_22_), ‘Pico Colorado’ (S_6_S_22_), ‘Rita’ (*S*_5_ *S*_22_)	OQ511572	EF429142	100.0	*P. avium*, *S*-RNase *S*_22_, complete cds
***S*_53_**	418	424		6		OQ511589	AB364468	80.0	*P. mume*, S-RNase S7, complete cds
***S*_19_**	421	427		6	‘Pflugwirtle B 12’ (*S*_7_ *S*_19_)	OQ511571	GU968641^b^	95.7^b^	*P. pseudocerasus*, *S*-RNase *S*_11_, complete cds
***S*_6_**	436	443		7	‘Ferobi’ (*S*_6_ *S*_9_), ‘Fertard’ (*S*_3_ *S*_6_), ‘Pico Colorado’ (S_6_S_22_)	OQ511566	EU077236	100.0	*P. avium*, *S*-RNase *S*_6_, partial cds
***S*_4_**	445	451		6	‘Feria’ (*S*_2_ *S*_4_), ‘Sir Don’ (*S*_4_ *S*_13_)	OQ511564	AJ635288	100.0	*P. avium*, *S-*RNase *S*_4_, exons 1-2
***S*_4’_**	445	451		6	‘Grace Star’ (*S*_4’_ *S*_9_), ‘Lapins’ (*S*_1_ *S*_4’_)				
***S*_13_**	n.a.	n.a.			‘Sir Don’ (*S*_4_ *S*_13_), ‘Sir Tom’ (*S*_3_ *S*_13_)				

^1^cultivars with known *S*-alleles used as reference for genotyping with ABI system.

a additional observed allele lengths with less frequency in the data set.

b no reference sequence (complete cds, exon 1-2) of *S_17_, S_18_, S_19_, S_32_* and *S_42_* in NCBI database available.

n.a, no amplification; n.d., not determined.

For each *S*-allele, the fragment length of the PCR product amplified with the PaConsI-F/R2 primer combination and determined with the ABI and Beckman systems, is given in ascending order. Cultivars with known *S*-alleles that were used as reference genotypes and analyzed on the ABI system are shown. In addition, amplicon deep sequencing of PCR products from selected individuals (indicated in **Supplementary Table S1**) was performed and the sequence obtained (Seq-ID) was compared with the PaConsI-F/R2 amplicon length. The best hit from the BLASTn analysis of the sequence is given, including the percentage of sequence identity and a short description. Red numbers indicate allele lengths that may be critical for accurate *S*-allele genotyping.

**Table 2:** Reference collection for *S*-allele genotyping established at JKI Dresden-Pillnitz.

Genotype	*S*-alleles	length [bp]		Reference collection	
				Ladder	Accession nr.
‘Adriana’	***S_1_S_14_***	374	325	x	KIZ-KIA0174
‘Alex’	***S_3_S_3’_***	229	229	–	KIZA04-19
‘Benjaminler’	***S_1_S_7_***	374	341	x	KIZA06-5
‘Feria’	***S_2_S_4_***	338	445	x	KIZA02-5
‘Ferobi’	***S_6_S_9_***	436	351	x	KIZA02-1
K18-200	***S_14_S_29_***	325	335	x	PiSue09-08.1
K18-204	***S_4_S_31_***	445	203	x	PiSue09-13.4
K18-218	***S_48_S_49_***	360	376	x	PiSue09-14.8
K18-233	***S_42_S_52_***	406	394	x	PiSue09-16.3
K18-235	***S_18_S_42_***	337	406	x	PiSue09-16.5
K18-245	***S_14_S_50_***	325	385	x	PiSue09-17.5
K18-302	***S_42_S_53_***	406	418	x	PiSue09-23.3
K18-339	***S_19_S_32_***	421	410	x	PiSue09-31.10
K18-343	***S_30_S_51_***	379	388	x	PiSue09-32.4
K18-346	***S_51_S_17_***	388	391	x	PiSue09-32.8
K18-361	***S_4_S_46_***	445	344	x	PiSue09-11.3
K18-362	***S_1_S_45_***	374	334	x	PiSue09-11.4
K18-402	***S_1_S_36b2_***	374	365	x	PiSue15-68.1
K18-415	***S_5’_S_36b3_***	386	363	x	PiSue15-73.5
K18-447	***S_4_S_47_***	445	345	x	PiSue15-82.4
K18-469	***S_14_S_34_***	325	372	x	PiSue15-87.2
‘Lapins’	***S_1_S_4’_***	374	445	–	KIZA07-5
‘Maergelikirsche’	***S_5_S_21_***	369	387	–	KIA1071
‘Nordwunder’	***S_3_S_12_***	229	339	x	KIZ-KIA0075
‘Pflugwirtle B 12’	***S_7_S_19_***	341	421	x	KIZ-KIA1051
‘Rita’	***S_5_S_22_***	387	417	x	KIZA04-9
‘Talaguera Brilante’	***S_21_S_22_***	369	417	x	–

The genotypes used for the *S*-allele ladder are indicated. The indicated fragment length of the PaConsI-F/R2 PCR amplicon refers to the analysis on the ABI instrument.

### 2.2 DNA extraction and *S-*allele genotyping

The total genomic DNA was extracted from peeled flower or leaf buds (65 – 85 mg) or leaf material (60 - 70 mg) using the DNeasy Plant Mini Kit (QIAGEN GmbH, Hilden, Germany) according to the manufactures protocol. After DNA quantification using the NanoDrop 2000c (ThermoFisher Scientific, Germany), DNA concentration was adjusted to 10 ng/µL in ddH_2_O. The primer set PaConsI-F/PaConsI-R2 (Sonneveld et al., 2003, Sonneveld et al., 2006) was used for amplification of the first intron of the *S-RNase* gene. The primer PaConsI-R2 was labeled at 5’-end with 6-FAM (for analysis with the ABI system) or with BMN-5 (for measurements with the Beckman system), both supplied by Biomers.net GmbH (Ulm, Germany). The PCR was performed in a final volume of 6 to 10 µL using the Type-it Microsatellite PCR Kit (QIAGEN GmbH, Hilden, Germany) adding the Q-solution with following PCR conditions: initial denaturation at 94°C for 2 min; 35 cycles of 94°C for 1 min, 54°C for 1 min and 72°C for 1 min; and a final elongation step at 60°C for 30 min.

For fragment length analysis with the ABI system, the PCR reaction was diluted with ddH_2_O in the ratio 1:100 or 1:200 in final volume of 200 µL, supplemented with 8.95 µL Applied Biosystems Hi-Di Formamid (Thermo Fisher Scientific Inc., USA) and 0.05 µL Applied Biosystems GeneScan 600 LIZ dye Size Standard v2.0 (Thermo Fisher Scientific Inc., USA). PCR fragment length determination was performed after denaturation at 95°C for 5 min on the ABI Genetic Analyzer 3500xl system (Applied Biosystems^®^ ThermoFisher Scientific, Germany) using the POP-7 Polymer (Thermo Fisher Scientific Inc., USA) and analysis was done with the Applied Biosystems GeneMapper Software 6.

For analysis with the Beckman system, the PCR reaction was diluted 1:20 with ddH_2_O and 2 μL was added to a mixture consisting of 24.9 μL sample loading solution and 0.1 μL DNA size standard 600 (Beckman Coulter, Krefeld, Germany). To prevent evaporation, a drop of mineral oil was added to the sample. Fragment length analysis was performed on the Beckman Coulter CEQ™ 8000 Genetic Analysis system (Beckman Coulter, Krefeld, Germany) equipped with a GenomeLab 33–75 separation capillary array (Beckman Coulter) and sizes were scored with the fragment analysis tool of the GenomeLab GeXP software (Beckman Coulter).

Allelic richness and private allelic richness were calculated for the *S-*locus using the Allelic Diversity Analyzer Version 1.0 (Szpiech et al., 2008). The maximal standardized sample size (MAX_G) was set to 50.

### 2.3 Ultra-deep sequencing of *S*-allele amplicons

The first intron of *S*-alleles was amplified using the unlabeled primer set PaConsI-F/PaConsI-R2 with the Thermo Scientific™ Phusion High-Fidelity PCR Kit (Thermo Fisher Scientific Inc., USA) according to the manufactures protocol. The PCR was performed in a final volume of 50 µL using 20 ng genomic template DNA and 1x HF buffer with following PCR conditions: initial denaturation at 98°C for 30 sec; 30 cycles of 98°C for 10 sec, annealing 58°C (optimized by gradient PCR) for 30 sec and 72°C for 30 sec; and a final elongation step at 72°C for 7 min. The PCR products were purified using the Thermo Scientific™ GeneJET PCR Purification Kit (Thermo Fisher Scientific Inc., USA) or the PCR Kleen™ Purification Spin Columns (Bio-Rad Laboratories GmbH, Germany) according to the manufacturer protocols, eluted with ddH_2_O. DNA quantification was done using the NanoDrop 2000c (Thermo Fisher Scientific, Germany). GENEWIZ Germany GmbH (Leipzig, Germany) provided amplicon ultra-deep sequencing on the Illumina platform (2x250 bp configuration) as well as unique sequence identification. The Basic Local Alignment Search Tool (BLAST) was used to find high similar sequences in the nucleotide collection of the National Center for Biotechnology Information (NCBI, https://www.ncbi.nlm.nih.gov/, last access 22.09.2020) for each identified unique sequence with a portion ≥ 1% of total reads. The Qiagen CLC Main Workbench 21.0.1 (Qiagen GmbH, Hilden, Germany) was used to create the alignment and the phylogenetic trees.

## 3 Results

### 3.1 Collection of sweet cherry genotypes from the region south of the Caucasus

**Figure 1:**
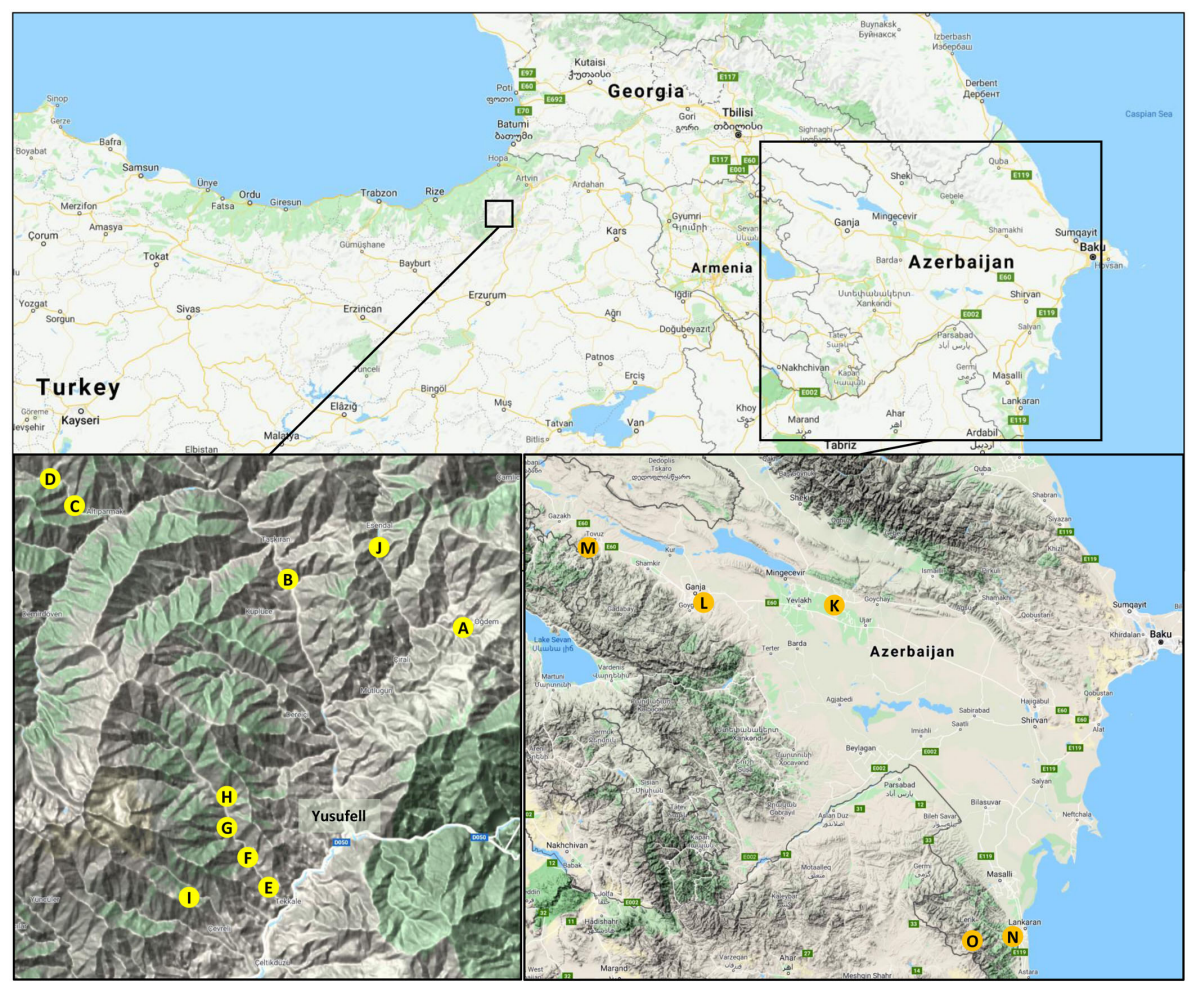
Collection areas of sweet cherry stones south of the Caucasus. The upper map gives an overview of the Caucasus region between the Black Sea and the Caspian Sea. The collection areas in Turkey and Azerbaijan, both located in the South Caucasus (Lesser Caucasus Mountains), are indicated by the squares. In the lower panels, the different collection sites are indicated by letters (left: Turkey, A-J; right: Azerbaijan, K-O). In the following, the number of genotypes analyzed is given in the form “collection site (number of mother trees, number of seedlings)”. A (1, 1); B (4, 40); C (1, 10); D (2, 20), E (1, 10); F (3, 30); G (2, 19); H (1, 10); I (2, 19); J (1, 4); K (3, 5), L (2, 6); M (6, 23); N (4, 19); O (9, 44). Detailed information on the plant material collected is available in **Supplementary Table S1**. (map material: Google Maps, 2020).

In June 2008, stones from 18 trees of local sweet cherry cultivars or wild sweet cherries (**Supplementary Table S1**) were collected in Turkey at an altitude of 800 – 1,600 m above sea level in three side valleys of the Çoruh River near the town of Yusufeli (**Figure 1**, sites A-J).

In 2014, stones from local cultivars and wild sweet cherries were collected in Azerbaijan, respectively. Stones from 11 genotypes were collected in the central region in Akdash, Ganja, and Tovus. Further 13 samples were taken in Lankaran and the Talish Mountains in the southern region near the border with Iran. Each sample consisted of several stones of each genotype. The stones were stratified and grown as described, and one to ten individual seedlings per mother tree were obtained. A total of 97 genotypes originating from Azerbaijan and 163 genotypes with an origin from Turkey were received and cultivated in the experimental field of the Julius Kühn-Institut in Dresden, Germany.

### 3.2 *S*-allele genotyping combining on PCR fragment length analysis and amplicon deep sequencing

The first intron of the *S-RNase* gene was amplified from each analyzed genotype using the primer pair PaConsI-F/R2 (Sonneveld et al., 2006). The lengths of the obtained fragments were determined and compared with two different capillary gel electrophoresis devices from the manufacturers ABI and Beckman (**Table 1**). For the assignment of the measured fragment lengths to already described *S*-alleles, several cultivars with known *S*-alleles (Schuster, 2017) were selected, analyzed on the ABI system and used as reference. For each of these *S*-alleles, one to five different cultivars served as independent references for genotyping. The *S*-allele genotyping results from each tree originating from stones collected in Azerbaijan and Turkey are presented in **Supplementary Table S1**. Comparing both analytical instruments, the determined fragment lengths differed in most cases by 5 to 6 bp. In addition to the fragment lengths assignable to *S*-alleles *S*_1_, *S*_2_, *S*_3_/*S*_3’_, *S*_4_/*S*_4’_, *S*_5_, *S*_6_, *S*_7_, *S*_9_, *S*_12_, *S*_14_, *S*_19,_ *S*_21_, and *S*_22_, further fragment lengths were observed when analyzing the collected material from the Caucasian region. For these fragments no clear *S*-allele assignment using the reference genotypes was possible. Therefore, amplicon deep sequencing of the PaConsI-F/R2 PCR products from selected individuals (**Supplementary Table S1**) was performed. The lengths of the sequences obtained from the sequencing approach correlated strongly with the values measured by fragment length analysis of the PCR products. BLASTn analysis of some sequences resulted in alignments with high levels of sequence identity (nearly 100%) with known *S*-allele sequences deposited in the NCBI database (**Table 1**). Using these sequence analyses, the *S*-alleles *S_5’_*, *S_29_*, *S_30_*, *S_31_*, *S_34_*, *S_36b2_*, *S_36b3_* could be linked to observed PCR fragment lengths. Further assignments to previously known *S-*alleles *S_17_*, *S_18_*, *S_32_*, and *S_42_* were made by comparing fragment lengths described in the literature (Vaughan et al., 2008; Cachi et al., 2017). For these alleles, no reference sequence of the corresponding region of the *S-RNase* gene was available in the NCBI database.

### 3.3 Newly identified *S*-alleles

Nine different PCR fragments were amplified that could not be assigned to any of the previously described *S-*alleles either based on their fragment length or on the basis of BLAST analyses using the amplicon sequence. According to the *S*-allele nomenclature, these newly identified *S-*alleles have been named, in ascending order, *S_45_* (334 bp), *S_46_* (344 bp), *S_47_* (345 bp), *S_48_* (360 bp), *S_49_* (376 bp), *S_50_* (385 bp), *S_51_* (388 bp), *S_52_* (394 bp) and *S_53_* (418 bp). The given fragment lengths refer to the measurements performed on the ABI instrument. Sequence alignments (**Supplementary Figure S1**) and a phylogram (**Supplementary Figure S2**) were generated with all the sequences obtained and a distinctness between the various *S*-allele sequences could be demonstrated. Particular attention should be paid to the *S*-allele sequences that are very similar in fragment length (**Table 1**). The newly defined alleles *S_46_* and *S_47_* differ by only one base pair in their fragment length using the ABI instrument. Using the Beckman system, an identical fragment length of 350 bp was determined for both alleles. However, the sequence alignment of both *S-*alleles revealed only 54% sequence identity and significant differences including large sequence gaps/insertions and substitutions (**Supplementary Figure S1**). A high degree of genetic divergence is also shown in the phylogram, as both sequences appear in distant branches (**Supplementary Figure S2**). This finding is also evident for other *S*-alleles (**Table 1**). Sequence differences between *S*-alleles with similar fragment lengths were also found for the newly identified *S*-alleles *S_45_* and *S_29_*, *S_49_* and *S_1_*, *S_50_* and *S_5’_*, *S_51_* and *S_5_* (NCBI sequence accession AJ635290), as well as *S_53_* and *S_22_*.

### 3.4 *S*-allele reference ladder as a robust tool for genotyping

**Figure 2:**
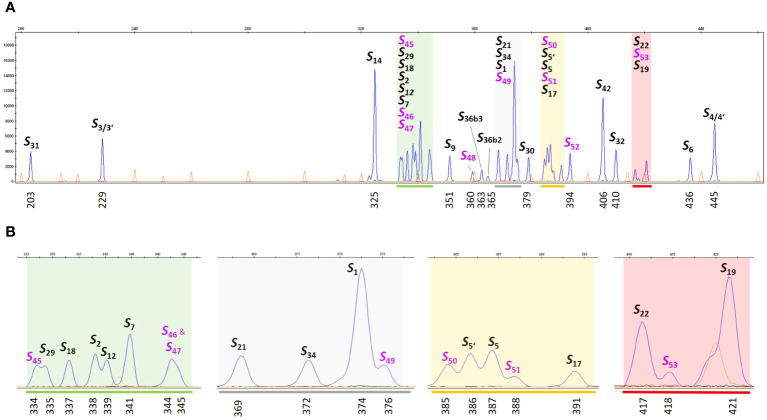
*S*-allele reference ladder for genotyping. *S*-allele specific fragments were amplified by PCR from reference genotypes (**Table 2**) using a FAM-labeled primer pair PaConsI-F/PaConsI-R2 flanking the first intron of the *S-RNase* gene. An *S-*allele reference ladder sample combining each PCR reaction was analyzed on the ABI Genetic Analyzer 3500xl system. **(A)** The diagram displays all *S-*allele specific peaks (blue) in relation to the size standard (orange). The calculated size of the fragment and the assigned *S-*allele is labeled (*S*-alleles, which were newly defined in this work are lettered in violet). **(B)** For better resolution, the colored areas in **(A)**, are displayed with a higher zoom factor.

The large number of *S*-alleles studied in this work shows that the fragment lengths of different alleles are sometimes very close to each other. The high density of marker alleles requires the use of suitable references for matching. To create a reference collection for *S*-allele genotyping, different genotypes with known *S*-alleles were selected to represent each *S*-allele at least once. These genotypes were planted in the experimental field of the Julius Kühn-Institute in Dresden, Germany (**Table 2**), where they will be maintained for future studies. To make the use of such a large number of references in capillary gel electrophoresis simple and practical, an *S*-allele reference ladder was developed, consisting of a mixture of PCR products amplified from selected reference genotypes (**Table 2**). The resulting chromatogram of this reference ladder is shown in **Figure 2**. Delineated peaks allow accurate genotyping of 31 different *S-*alleles. In addition, the alleles *S_46_* and *S_47_* form a common broad peak in the pooled reference sample. In the respective individual samples, the peaks at 344 bp and 345 bp are clearly distinguishable from each other (not shown). Differentiation between *S_3_* and *S_3_*_’_ as well as *S_4_* and *S_4’_* is not possible with this analytical method. The *S*-allele reference ladder can be used as a single standard sample to calibrate run-to-run and lab-to-lab differences on any capillary electrophoresis instrument. This increases the robustness of *S*-allele genotyping in future studies.

### 3.5 Distribution of *S*-alleles among the genetic resources originated from the Caucasus region

**Figure 3:**
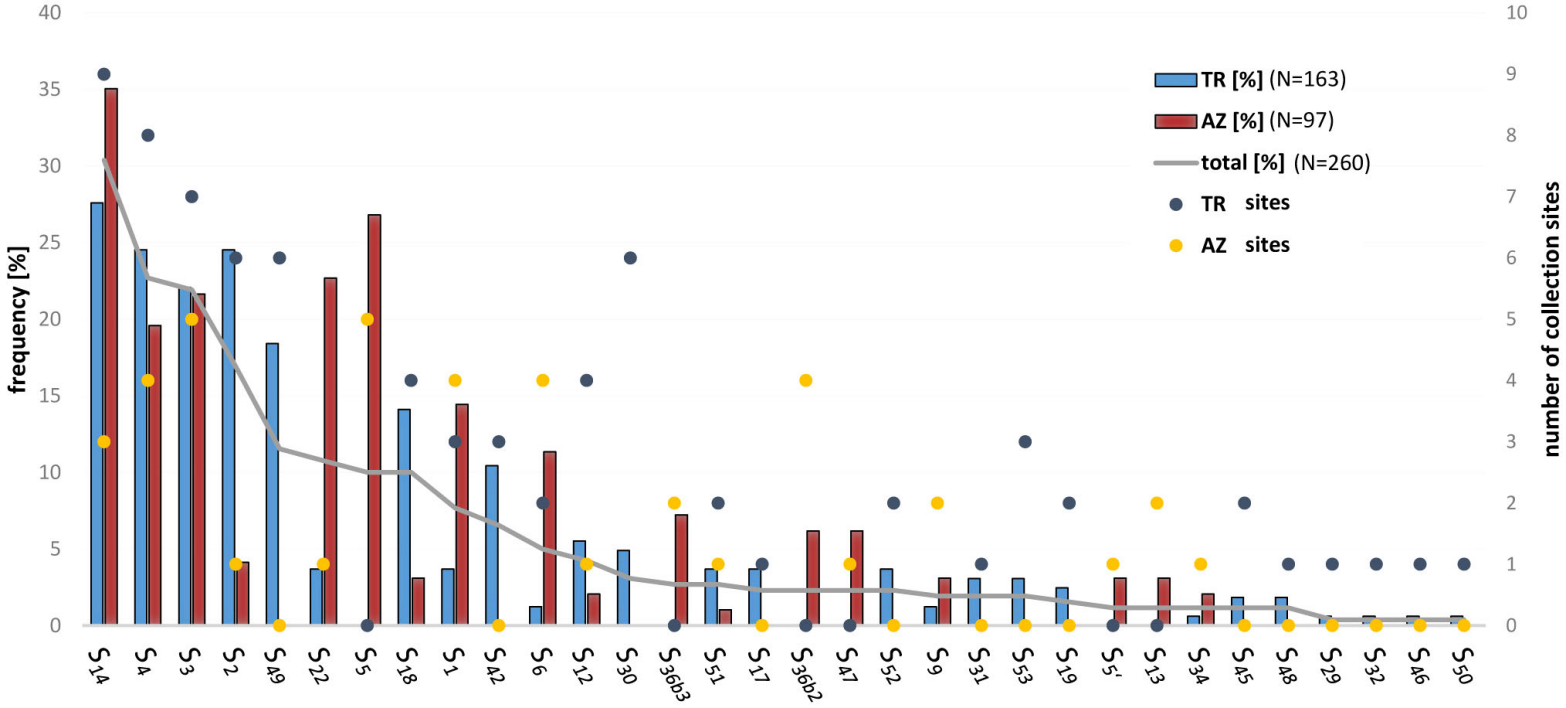
Frequency and distribution of single *S-*alleles in the collection area south of the Caucasus. The percentage frequency of *S-*alleles in the collected sweet cherries is shown as a grey line in descending order. The percentage proportion of *S-*alleles in the progeny with origin in Turkey (TR) and Azerbaijan (AZ) is represented by columns. The colored dots represent the total number of collection sites where this *S-*allele was detected (in total 10 sites in TR and 5 in AZ).

Among the genotypes originated from sweet cherry stones collected in the Caucasus region, 32 different *S-*alleles were observed. An overview of the distribution and frequency of these alleles in Turkey and Azerbaijan is given in **Figure 3**; **Table 3**. Twelve *S*-alleles were detected in progeny from both countries. Fourteen *S-*alleles were found only in sweet cherries originating from Turkey, whereas six *S-*alleles were found only in cherries from Azerbaijan. Most of the newly identified *S-*alleles, namely *S_45_*, *S_46_*, *S_48_*, *S_49_*, *S_50_*, *S_52_*, and *S_53_* were found only in cherries from Turkey. Among them, the *S_49_* allele was frequently observed, with a proportion of 18% in all progeny and a wide distribution in the Turkish collection region (present in six out of ten collection sites) (**Supplementary Table S2**). The remaining alleles were found at much lower frequencies, in most cases only at one or two collection sites (**Table 3**; **Supplementary Table S2**). *S_47_* was found only in cherries from Azerbaijan with a frequency of 6% and *S_51_* was detectable in plants from both countries with a low abundance (4% in the Turkish collection, 1% in the Azerbaijan collection). The most frequent allele in cherries from both countries was *S_14_*, followed by *S_4_* and *S_3_*, which were also found in cherries from both countries (**Figure 3**). In addition, the alleles *S_2_*, *S_49_*, *S_22_*, and *S_5_* also show high frequencies, but with a clear correlation with the collection region. *S_2_* and *S_49_* are characteristic of the Turkish material and *S_22_* and *S_5_* occur mainly in the material collected in Azerbaijan.

**Table 3:** Occurrence and distribution of *S*-alleles in genetic resources originating from Caucasian sweet cherries.

Collected in country	Turkey		Azerbaijan	
**Sampled donor trees**	17		24	
**Analyzed individuals of the progeny**	163		97	
**Detected *S*-alleles**	26		18	
**common for both countries**	12			
	*S_1_*, *S_2_*, *S_3_*, *S_4_*, *S_6_*, *S_9_*, *S_12_*, *S_14_*, *S_18_*, *S_22_*, *S_34_*, *S_51_*			
**Unique for country**	14	*S_17_*, *S_19_*, *S_29_*, *S_30_*, *S_31_*, *S_32_*, *S_42_*, *S_45_*, *S_46_*, *S_48_*, *S_49_*, *S_50_*, *S_52_*, *S_53_*	6	*S_5_*, *S_5’_*, *S_13_*, *S_36b2_*, *S_36b3_*, *S_47_*
**Frequent *S*-alleles^1^**	7	*S_2_*, *S_3_*, *S_4_*, *S_14_*, *S_18_*, *S_42_*, *S_49_*	7	*S_1_*, *S_3_*, *S_4_*, *S_5_*, *S_6_*, *S_14_*, *S_22_*
**Occurrence at single collection sites**	9	*S_17_* (I), *S_22_* (B), *S_29_* (A), *S_31_* (B), *S_32_* (H), *S_34_* (D), *S_46_* (J), *S_48_* (B), *S_50_* (D)	8	*S_2_* (N), *S_5’_* (M), *S_12_* (L), *S_18_* (N), *S_22_* (O), *S_34_* (O), *S_47_* (N), *S_51_* (M)
**Stray find**	5	*S_29_* (A), *S_32_* (H), *S_34_* (D), *S_46_* (J), *S_50_* (D)	1	*S_51_* (M)

^1^found in more than 10% of the analyzed individuals.

The total number of different *S*-alleles is given and these are specified for the respective category. The indicated letters refer to the collection sites defined in **Figure 1**.

One hundred and six *S*-genotypes were be detected in total. The frequency of each *S*-allele genotype and its distribution in the collection material according to its origin is shown in **Supplementary Table S3**. There are 74 different *S*-allele combinations present in cherries from Azerbaijan and 39 in cherries from Turkey. Only seven *S*-allele combinations (7%) were found in plants from both countries, while the other genotypes were found in only one country.

### 3.6 High *S*-allele diversity in sweet cherries collected in the Caucasus

**Figure 4:**
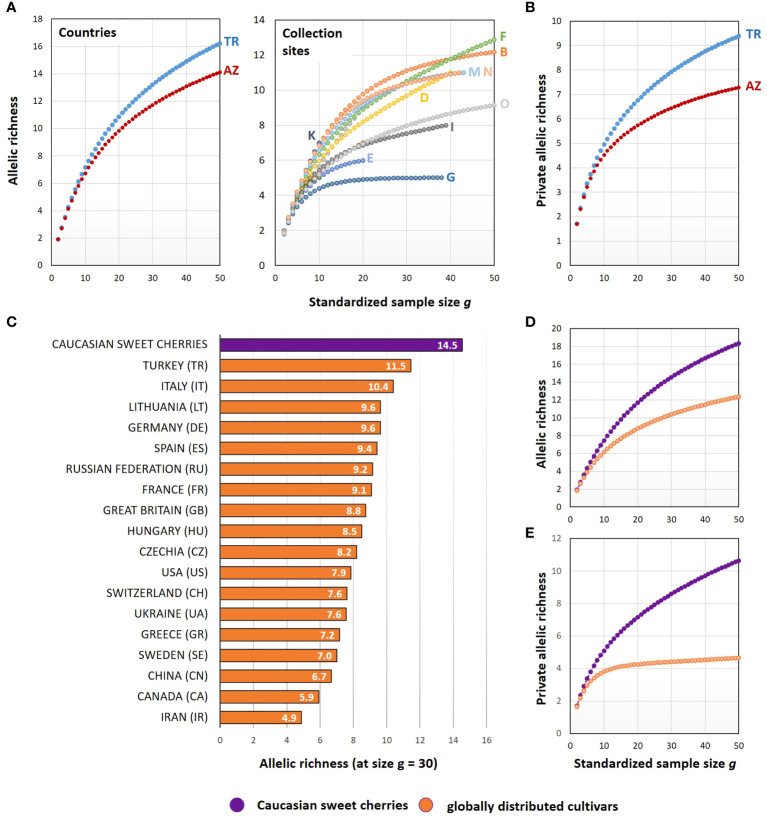
Comparison of the *S*-allele diversity of sweet cherries collected in the Caucasus and sweet cherry cultivars distributed worldwide. Allelic richness and the private allelic richness were calculated for the sweet cherries collected in Turkey and Azerbaijan and for sweet cherry cultivars originating from different countries. For this comparison, only countries with at least 30 cultivars for which the *S*-alleles are known were considered. The information on the *S-*alleles of these cultivars comes from (Schuster, 2020) **(A, B)** The grouping of the sweet cherries collected in the Caucasus was done for different countries (TR: Turkey; AZ: Azerbaijan) and collection sites (A–O, see **Figure 1**). **(C)** The allelic richness of the *S*-locus is presented for the sweet cherries collected in the Caucasus and for sweet cherry cultivars distributed worldwide, grouped by their country of origin. **(D, E)** Comparison of all cultivated sweet cherries and the sweet cherries collected in the Caucasus.

To study the *S*-allele diversity of the collected material, the number of distinct alleles (allelic richness) and the number of private alleles (private allelic richness) were calculated and normalized to a standardized sample size (**Figure 4**). The cherries from Turkey exhibited a higher allelic richness compared to cherries from Azerbaijan (**Figure 4A**). This was also the case for the number of private alleles (**Figure 4B**). Comparing the allelic richness of the distinct collection sites (**Figure 4A**), a high degree of variability was observed, with sites B and F (both in Turkey) showing the highest allelic richness.

The genetic diversity of the *S*-locus was also analyzed for cultivated sweet cherries of different countries. Therefore, data previously published and regularly updated by (Schuster, 2020) were used. This data set contains the *S*-genotypes of 2,966 sweet cherry cultivars including information to the respective country of origin. The sweet cherries collected in the Caucasus exhibited a higher allelic richness (**Figure 4D**) compared to the sweet cherry cultivars listed by (Schuster, 2020). This was also the case for the private allelic richness (**Figure 4E**). With increasing sample size g, the number of private alleles is steadily increasing calculated for the sweet cherries from the Caucasus, reaching a value of about 11 private alleles at sample size g=50. In contrast, the number of private alleles stagnates for the group of sweet cherry cultivars and reaches only a value of about five.

A more detailed view to the genetic diversity of the *S*-locus of cultivated sweet cherries grouped to their country of origin and compared to the Caucasian sweet cherries is represented in **Figure 4C**. The highest allelic richness was found in the Caucasian cherries. They showed a clear distance to the remaining sweet cherry cultivars. The Caucasian cherries were followed by cultivars from Turkey, Italy, Lithuania, Germany and Spain. The lowest allelic richness was found in cultivars from Iran, followed by Canada and China.

In summary, a high degree of allelic richness of the *S*-locus was observed for the plants originating from seeds of wild sweet cherries and local sweet cherry cultivars, which were collected in only a limited area in the Caucasian center of origin. In this regard, the comparison with the large group of worldwide-distributed sweet cherry cultivars reflected the reduction of genetic diversity of this fruit crop by selective breeding.

## 4 Discussion

### 4.1 Reference genotypes and amplicon-deep sequencing for *S*-allele genotyping

Determining *S*-alleles in sweet cherries is important for plant breeding and fruit production, as it allows for the selection of compatible combinations of *S*-genotypes successful pollination. The development of lab methods for predicting the compatibility of genotypes, which eliminates the need for elaborate test crosses, gained significant importance about 30 years ago. Initially, biochemical methods were developed using protein extracts obtained from style tissue to determine *S*-alleles based on the *S-RNase* polymorphism (Bošković and Tobutt, 1996; Bošković et al., 1997). A major simplification of *S*-genotyping was achieved by using PCR-based methods relying on the amplification of DNA length polymorphisms within the *S*-locus and the development of allele-specific primer combinations (Sonneveld et al., 2001, Sonneveld et al., 2003, Sonneveld et al., 2006).

Based on the results obtained in these initial studies, a consensus primer pair PaConsI-F/PaConsI-R2 was developed (Sonneveld et al., 2006) that binds specifically to conserved regions flanking the first intron of the *S-RNase* gene. This primer pair has since been used as a standard for the determination of *S*-alleles. Due to the large literature reference database developed with the PaConsI-F/R2 primer combination, this marker was also used in this work. However, the exact assignment of *S*-alleles remained difficult due to variations in analysis parameters such as different methods for separating amplicons (e.g. agarose gel versus capillary gel electrophoresis), types of devices for capillary gel electrophoresis (e.g. Beckman Coulter CEQ 8000, Thermofisher 3500xL Genetic Analyzer), and fluorescent dyes used for labeling primers. These differences can influence measured values in fragment length determination and cause system-related size shifts. This can also be proven by the data measured in this work. A size shift of 5 to 6 bp between the two different capillary electrophoresis devices used could be detected (**Table 1**, **Supplementary Table S1**). Further, the steadily increasing number of identified *S*-alleles, some of which differ in fragment length by only one or a few bases, exacerbates this problem. Examples are the *S_2_*, *S_7_* and *S_12_* alleles (Sonneveld et al., 2006; Vaughan et al., 2006; Cachi et al., 2017). The use of reference genotypes with known *S*-alleles is essential for the exact assignment of amplicons of almost the same size and the normalization of system-related size shifts.

In this study, one to five reference genotypes were analyzed for each of the 16 previously described *S*-alleles *S*_1_, *S*_2_, *S*_3_, *S*_3’_, *S*_4_, *S*_4’_, *S*_5_, *S*_6_, *S*_7_, *S*_9_, *S*_12_, *S*_13_, *S*_14_, *S*_19,_ *S*_21_, and *S*_22_ to allow a reliable assignment of the amplified fragment lengths to the respective *S-*allele. Unfortunately, reference material for *S-*genotyping was not available for all the *S-*alleles already described. These include the *S*_5’_, *S*_17_, *S*_18_, *S*_29_, *S*_30_, *S*_31_, *S*_32_, and *S*_34_ alleles. Some of them have only been reported in wild cherry (Vaughan et al., 2008), whereas they have not yet been detected in sweet cherry cultivars. Examples are the alleles *S_29_*, *S_31_*, *S_32_*, and *S_34_* (Schuster, 2020).

Analysis of the cherry genotypes collected in Turkey and Azerbaijan revealed a large number of *S-*alleles whose amplicons could not be assigned to the already known *S*-alleles of the available reference genotypes due to their different fragment size. To clarify whether these amplicons originated from previously unknown *S*-alleles, these fragments were sequenced using Illumina ultra-deep sequencing. Sequence alignments of the obtained sequences with sequences available in public databases allowed their assignment to the *S*-alleles *S_5’_*, *S_29_*, *S_30_*, *S_31_*, *S_32_*, and *S_34_*. In this way, the assignment of several amplicons to previously described *S*-alleles could be verified and confirmed (**Table 1**). For *S_17_* and *S_18_*, no reference sequences covering the region of the first intron could be found in the database. Here, an assignment was made by comparison with literature values (Cachi et al., 2017), considering possible size shifts.

Together with the *S-*alleles newly described in this work, 36 different *S*-haplotypes were analyzed in this study. In order to facilitate future *S*-allele genotyping studies and to make them comparable, a reference set of genotypes was compiled. This reference set of genotypes has been planted in the JKI’s trial field so that material from these plants can be provided on request for future genotyping studies. The reference set is structured in such a way that each *S*-haplotype is included at least once (**Table 2**). Further genotypes can be continuously added to this reference set to fill in missing or newly identified *S*-alleles, such as the recently published *S_54_* allele from the variety ‘Techlovicka’ (Cmejlova et al., 2023).

### 4.2 High resolution of *S-*genotyping by *S*-allele reference ladder

To improve the comparability and assignment of amplicons to known *S*-alleles based on their fragment length, an *S-*allele reference ladder was developed (**Figure 2**). This reference ladder contains pooled PCR products amplified from all *S*-haplotypes previously distinguished by fragment length analysis. This ladder can be added as a single control sample to all future studies. This allows for rapid normalization of instrument-dependent size shifts and calibration of run-to-run differences. The ladder also allows discrimination between *S*-alleles with amplified fragment lengths that are very close to each other. The resolution has been shown to be very high. By using the *S-*allele reference ladder, even very close peaks could be clearly assigned to different *S*-alleles. For example, alleles *S_45_* (334 bp), *S_29_* (335 bp), *S_18_* (337 bp), *S_2_* (338 bp), *S_12_* (339 bp) and *S_7_* (341 bp), which are only one or two base pairs apart, form clearly distinguishable peaks in the *S*-allele reference ladder (**Figure 2**). Despite the high accuracy of the fragment length determination, a doubtless assignment to known *S-*alleles by a simple comparison with published fragment lengths is not possible due to the instrument-related size shifts of several base pairs. This is only possible by using suitable reference samples in each experiment.

However, this work also showed that fragment length alone is a weak criterion for the detection of a specific *S*-allele. Based on sequence comparisons of the newly described *S_46_* and *S_47_* alleles, amplicons of identical size are expected. However, both *S*-allele sequences have a low degree of sequence identity (**Supplementary Figure S1**) and are phylogenetically distant from each other (**Supplementary Figure S2**). While the Beckman system detected an identical fragment length for both *S*-alleles, the ABI system detected a minimal size shift between the two alleles. In case of doubt, sequencing or allele-specific PCRs should be performed.

### 4.3 Limitations of *S*-allele genotyping using the universal marker PaConsI-F/R2

For some alleles, the PaConsI-F/R2 primer pair does not allow reliable determination of the *S*-allele. Examples of this are the alleles S_3’_ and S_4’_. Both alleles derived from their ancestral alleles *S_3_* and *S_4_* by induced mutagenesis. In this process, irradiation generated deletions at the *S*-locus that lie outside the region flanked by the primers PaConsI-F/R2 (Lewis and Crowe, 1954; Sonneveld et al., 2005). Consequently, PCR fragment sizes amplified from *S_3_* and *S_3’_* as well as *S_4_* and *S_4’_* with PaConsI-F/R2 are identical to each other. Thus, no distinction can be made between the ancestral and the mutated allele using PaConsI-F/R2. This is only possible by a subsequent analysis with allele-specific primer pairs. This is not the case for another mutant *S-*allele, called S_5’_. This allele is of natural origin and occurs in Sicilian varieties such as ‘Kronio’ (Marchese et al., 2007). The PaConsI-F/R2 fragment amplified from the *S_5’_* allele differs from the ancestral *S_5_* allele by a deletion of one base pair. This difference can be detected by fragment length analysis and the use of the reference ladder.

Furthermore, the marker PaConsI-F/R2 is not suitable for the amplification from the *S_13_* allele as no PCR product or eventually a SSR like trace peak is produced, which has been already reported (Sonneveld et al., 2006; Marchese et al., 2010; Lisek et al., 2015) and confirmed in this work. Although the primer binding sites are present in S_13_, a short highly variable tandem repeat, presenting up to 25 (AT) repeats in *P. avium* and up to 38 in *P. cerasus* (Marchese et al., 2010) in the amplified region probably inhibits the PCR, as already discussed by Cmejlova et al. (2023). For three genotypes of the Caucasian collection, the allele *S_13_* was assigned on the basis that in these only one PCR product was detected and a diploid, self-incompatible genotype was assumed. It cannot be completely excluded that other *S* alleles, which cannot be amplified with PaConsIF/R2, are present in these few genotypes. For confirmation of the *S_13_* allele, fluorescently labelled allele-specific primers could be included in future studies, that amplify across the two SSR regions present in the first and second intron of the *S_13_ S-RNase* gene (Marchese et al., 2010).

The use of *S*-allele-specific primer pairs and other markers within the *S*-locus is an alternative to overcome the problems mentioned above. Recently, a new one-tube PCR assay with subsequent fragment analysis for *S* genotyping of *P. avium* has been developed (Cmejlova et al., 2023), based on multiplex PCR with 27 different primers, 8 of which are labeled with a fluorescent dye. This complex assay contains sequence-optimized primers for the universal amplification of the first intron of the *S-RNase* gene based on the PaConsI-F/R2 binding sites, a set of several *S-*allele specific primers, and a marker for the detection of *MGST* alleles.

### 4.4 Basis material with high level of diversity for breeding

In this work, nine previously undescribed *S-*alleles were identified in the collected genetic material from the Causcasus. Although, the collection area was limited to a small area in Turkey and Azerbaijan. It can be assumed that the genetic diversity in the area of origin of the sweet cherry is much greater and that the number of distinguishable *S*-alleles will increase with further investigations. This goes hand in hand with the increasing complexity in the application of *S*-allele genotyping. On the other hand, only a fraction of the *S-*allele diversity has been incorporated into varieties cultivated today. This is well demonstrated in this study by the comparison of allelic richness and private allelic richness between the analyzed material from the Caucasus and the globally distributed cultivars performed in this study (**Figure 4**). For the applicability of *S-*allele genotyping in the field of breeding and fruit production, the question arises to what extent the nomenclature of alleles by ascending numbering should also be maintained for original and wild material of the species *P. avium*. However, the newly established *S*-allele reference ladder in combination with amplicon deep sequencing considerably facilitate the determination of *S*-alleles. Both tools can now be used to determine new *S*-alleles and to identify preferred genotypes as part of marker-assisted selection.

Genetic diversity in cultivated sweet cherries is severely limited. Domestication has been described as a major bottleneck and breeding has further reduced diversity (Pinosio et al., 2020). For the breeding of sweet cherry cultivars with new traits adapted to changing environmental conditions, the low diversity in cultivated elite breeding material is a severe limitation. Collecting genetic resources in the region of origin of the species aims to obtain basic material characterized by a high diversity of traits of great interest for future breeding programs. In this study, it could be shown by means of *S-*allele genotyping that the genetic diversity in the collected material from the Caucasus is significantly higher than the diversity of globally distributed sweet cherries. It can be assumed that other traits with significance for breeding new, adapted varieties also show higher diversity. The use of these genetic resources in future breeding programs for the introduction of new traits is aimed at. Thus, the *S-*alleles, which have so far only been determined in basic material for breeding, should also be considered for future genotyping projects.

## 5 Conclusions

Based on the results of this study, the following conclusions can be drawn. The genetic diversity of the collection material from the center of origin appears to be significantly higher (at least on the basis of the *S*-alleles) than the diversity in the current spectrum of traditional and commercially grown sweet cherry cultivars. This seems to be due to the fact that originally only a few genotypes contributed to the development of the genepool of cultivated sweet cherries. Major progress and genetic gains through breeding can only be expected in the future if the diversity in the breeders’ gene pool is expanded. This can be achieved by collecting material in the center of origin and the continuous introgression of new traits into the breeding pool as part of pre-breeding programs. In addition, the areas of origin should be safeguarded through suitable protective measures.

## Data availability statement

The datasets presented in this study can be found in online repositories. The names of the repository/repositories and accession number(s) can be found in the article/**Supplementary Material**.

## Author contributions

SS: Conceptualization, Formal analysis, Investigation, Methodology, Visualization, Writing – original draft, Writing – review & editing. MS: Conceptualization, Resources, Writing – review & editing. JQ-G: Resources, Writing – review & editing. GL-O: Resources, Writing – review & editing. HF: Conceptualization, Funding acquisition, Writing – original draft, Writing – review & editing.
