# Supplementary material for: Synergistic approach of PCR-based fragment length analysis and amplicon deep sequencing reveals rich diversity of S-alleles in sweet cherries from the Caucasian region of origin
Source: Front Plant Sci. 2024 Apr 5;15:1355977. doi: 10.3389/fpls.2024.1355977 (PMC11067951; doi:10.3389/fpls.2024.1355977)
Supplement: Supplementary file 1 [file DataSheet_1.zip › Figure S1.PDF]

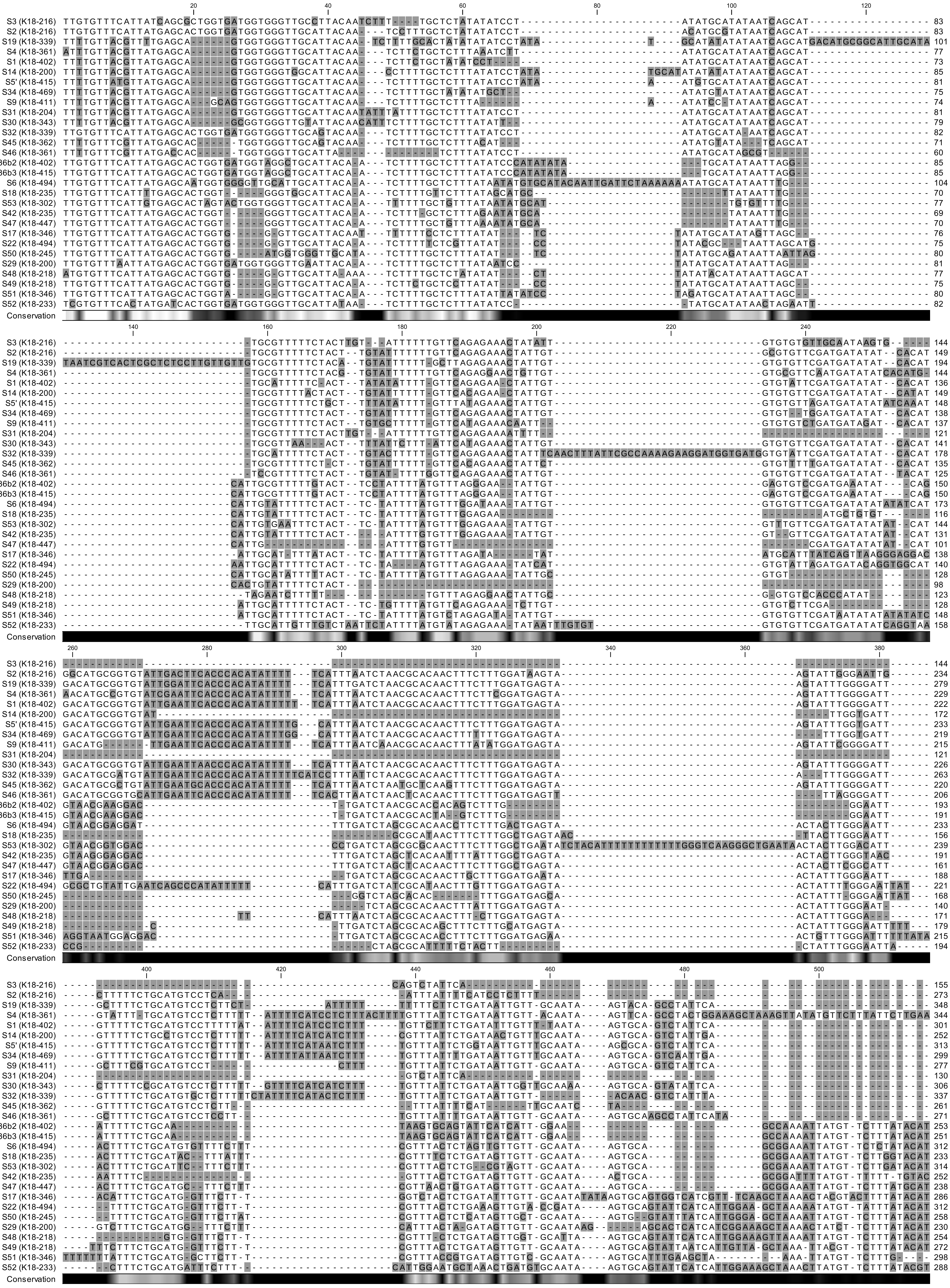
S3 (K18-216)
S2 (K18-216)
S19 (K18-339)
S4 (K18-361)
S1 (K18-402)
S14 (K18-200)
S5' (K18-415)
S34 (K18-469)
S9 (K18-411)
S31 (K18-204)
S30 (K18-343)
S32 (K18-339)
S45 (K18-362)
S46 (K18-361)
S36b2 (K18-402)
S36b3 (K18-415)
S6 (K18-494)
S18 (K18-235)
S53 (K18-302)
S42 (K18-235)
S47 (K18-447)
S17 (K18-346)
S22 (K18-494)
S50 (K18-245)
S29 (K18-200)
S48 (K18-218)
S49 (K18-218)
S51 (K18-346)
S52 (K18-233)
Conservation

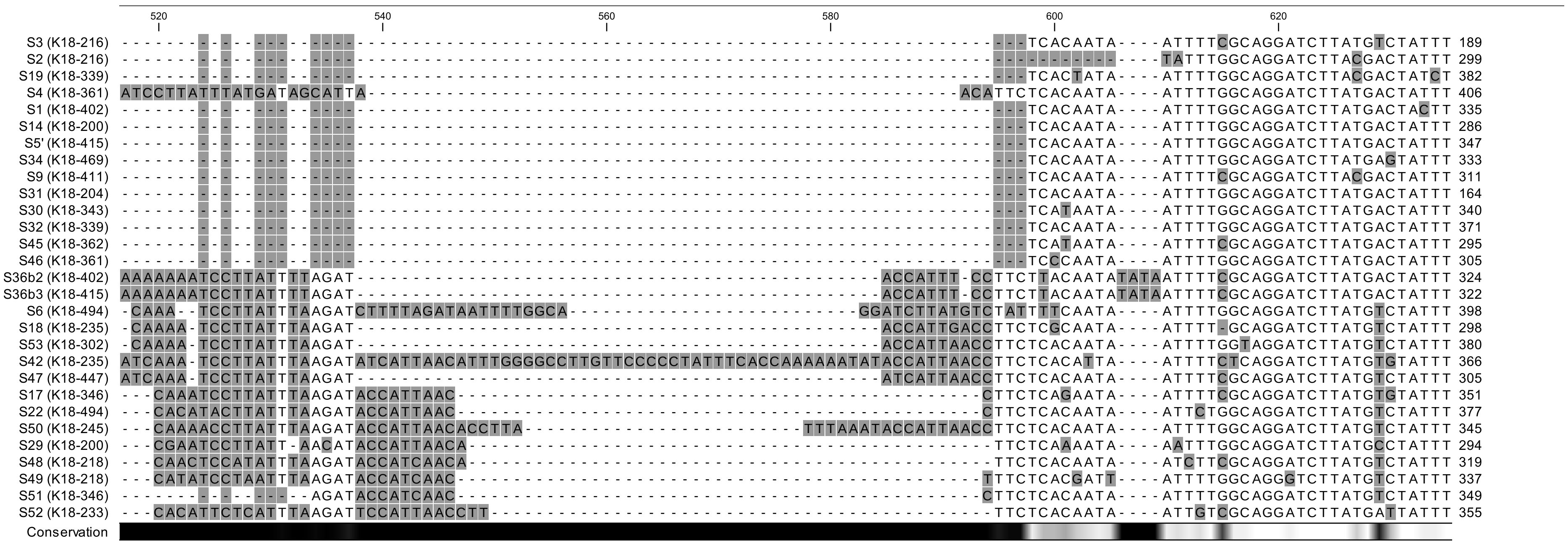

Figure S1: Alignment of S-allele sequences obtained by amplicon deep sequencing.

The region spanning the first intron of the *S-RNase* gene was amplified from different individuals of the sweet cherry collection (indicated in the brackets) and the PCR products were sequenced by amplicon deep sequencing. The alignment contain unique sequences assigned to each S-allele, sorted by similarity. Primer sequences up- and downstream of the sequences are trimmed. The degree of conservation is represented by greyscale (black/low – white/strong conservation). Alignment positions that differ from the consensus sequence are highlighted in grey.
