# Supplementary material for: Synergistic approach of PCR-based fragment length analysis and amplicon deep sequencing reveals rich diversity of S-alleles in sweet cherries from the Caucasian region of origin
Source: Front Plant Sci. 2024 Apr 5;15:1355977. doi: 10.3389/fpls.2024.1355977 (PMC11067951; doi:10.3389/fpls.2024.1355977)
Supplement: Supplementary file 1 [file DataSheet_1.zip › Figure S2.PDF]

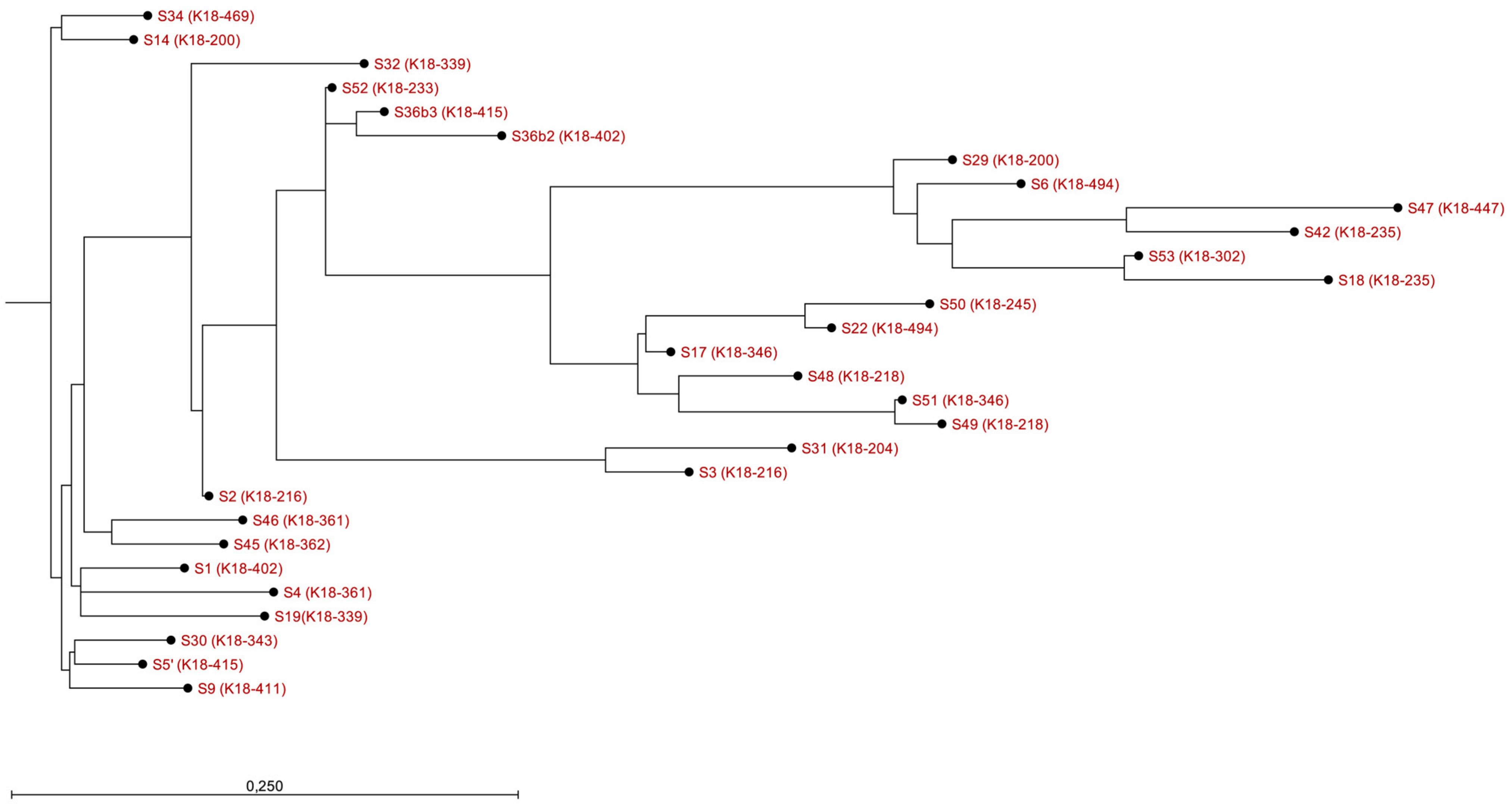

Figure S2: Phylogram of S-allele sequences.
Sequences containing the first intron of the *S-RNase* gene (Fig. S1) of each S-allele were used to construct the tree by Maximum Likelihood Phylogeny (construction method: Neighbor Joining; nucleotide substitution model: Jukes Cantor).
